# Supplementary material for: Van der Waals five-body size-energy universality
Source: Sci Rep. 2022 Jun 20;12:10368. doi: 10.1038/s41598-022-13630-2 (PMC9209460; doi:10.1038/s41598-022-13630-2)
Supplement: Supplementary file 1 — Supplementary Information. [file 41598_2022_13630_MOESM1_ESM.pdf]

Supplementary Information

Five-body size-energy universality

Petar Stipanović[1,*], Leandra Vranješ Markić[1] and Jordi Boronat[2]

[1]University of Split, Faculty of Science, Split, Croatia. [2]Departament de Física, Campus Nord B4-B5, Universitat Politècnica de Catalunya, Barcelona, Spain.

*e-mail: pero@pmfst.hr

Supplementary Table S1: The ground-state properties of the cluster A$_a$B$_b$C$_c$, predicted with given models of pair interactions $V(r)$: pair van der Waals length R, mean square pair size $\langle r^2\rangle$, ratio of the largest and the smallest pair size $\langle r^2\rangle_{\mathrm{Max}}/\langle r^2\rangle_{\mathrm{min}}$, binding energy $B = |E|$, number of self-bound sub-dimers N_2, scaled energy $X_E = mB\rho_R^2\hbar^{-2}$ and scaled size $Y_\rho = \rho_r^2\rho_R^{-2}$, where m is a mass unit, M is total mass, $\hbar$ is reduced Planck constant and $\rho_{r,R}^2$ squared hyperradius of lengths $\langle r^2\rangle$ and R^2, i.e. $\rho_r^2 = \frac{1}{Mm}\sum_{i<k}^{5} m_i m_k \langle r_{ik}^2\rangle$. Energies for clusters of helium and alkali isotopes have been published.[1] Energies for some models of ^{4}He$_n$^{3}H$\downarrow_{5-n}$ are evaluated for the first time, while for other models[2] are recalculated to reduce the variance. All other properties and clusters are analyzed for this work. The last digit of reported values is uncertain.

Cluster A$_a$B$_b$C$_c$						potential	R / Å						$\langle r^2\rangle$ / Å^2						$\langle r^2\rangle_{\mathrm{Max}}/$				
A	B	C	a	b	c	$V(r)$	A-A	B-B	C-C	A-B	A-C	B-C	A-A	B-B	C-C	A-B	A-C	B-C	$\langle r^2\rangle_{\mathrm{min}}$	B / mK	N_2	X_E	Y_ρ
^{4}He			5			V[3]	5.38						59.4						1.00	1334.9	10	6.37	2.05
^{4}He	^{3}He		4	1		V[3]	5.38			5.18			63			84			1.33	941.1	6	4.15	2.48
^{4}He	^{3}He		3	2		V[3]	5.38	5.01		5.18			70	119		95			1.70	600.0	3	2.44	3.18
^{4}He	^{3}H↓		4	1		V[3,4]	5.38			6.69			65			103			1.58	885.7	6	4.74	2.27
^{4}He	^{3}H↓		4	1		V[3,5]	5.38			6.10			65			106			1.63	866.9	6	4.30	2.48
^{4}He	^{3}H↓		4	1		V[3,6]	5.38			5.75			66			121			1.83	791.4	6	3.75	2.79
^{4}He	^{3}H↓		4	1		V[3,7]	5.38			6.10			67			172			2.57	682.8	6	3.39	3.22
^{4}He	^{3}H↓		4	1		V[3,8]	5.38			6.10			67			203			3.03	662.8	6	3.29	3.55
^{4}He	^{3}H↓		3	2		V[3,4,9]	5.38	7.28		6.69			75	139		111			1.85	577.5	3	3.38	2.52
^{4}He	^{3}H↓		3	2		V[3,5,9]	5.38	7.28		6.10			76.5	144		117			1.88	551.0	3	2.88	2.95
^{4}He	^{3}H↓		3	2		V[3,6,9]	5.38	7.28		5.75			80	163		131			2.04	441.6	3	2.15	3.48
^{4}He	^{3}H↓		3	2		V[3,7,9]	5.38	7.28		6.10			89	227		183			2.55	287.5	3	1.50	4.29
^{4}He	^{3}H↓		3	2		V[3,8,9]	5.38	7.28		6.10			93	265		214			2.85	256.0	3	1.34	4.90
^{4}He	^{3}H↓		2	3		V[3,4,9]	5.38	7.28		6.69			93	147		127			1.58	408.8	1	2.55	2.85
^{4}He	^{3}H↓		2	3		V[3,5,9]	5.38	7.28		6.10			96	150		130			1.56	384.4	1	2.14	3.27
^{4}He	^{3}H↓		2	3		V[3,6,9]	5.38	7.28		5.75			110	167		151			1.52	284.3	1	1.48	4.01
^{4}He	^{3}H↓		2	3		V[3,7,9]	5.38	7.28		6.10			152	217		216			1.43	146.0	1	0.81	5.21
^{4}He	^{3}H↓		2	3		V[3,8,9]	5.38	7.28		6.10			175	240		246			1.41	117.0	1	0.65	5.90
^{4}He	^{3}H↓		1	4		V[4,9]		7.28		6.69				156		144			1.08	358.7	0	2.33	3.06
^{4}He	^{3}H↓		1	4		V[5,9]		7.28		6.10				158		145			1.09	344.4	0	2.07	3.33
^{4}He	^{3}H↓		1	4		V[6,9]		7.28		5.75				166		170			1.02	283.4	0	1.63	3.85
^{4}He	^{3}H↓		1	4		V[7,9]		7.28		6.10				188		240			1.28	202.0	0	1.21	4.66
^{4}He	^{3}H↓		1	4		V[8,9]		7.28		6.10				193		290			1.50	184.5	0	1.11	5.23
	^{3}H↓			5		V[9]		7.28						158.5					1.00	399.0	0	2.63	2.99
^{4}He		^{2}H↓	4		1	V[3,4]	5.38	5.01			5.73		68				370		5.44	612	6	2.69	4.81
^{4}He		^{2}H↓	3		2	V[3,4,9]	5.38		6.58		5.73		106		1900		940		17.9	143	3	0.58	18.5
^{4}He	^{3}He	^{3}H↓	3	1	1	V[3,4]	5.38			5.18	6.10	5.91	72			99	114	138	1.92	560	3	2.58	3.09
^{4}He	^{3}He	^{2}H↓	3	1	1	V[3,4]	5.38			5.18	5.73	5.59	80			111	600	632	7.90	331	3	1.34	7.84
^{4}He	^{3}He	^{3}H↓	2	2	1	V[3,4]	5.38	5.01		5.18	6.10	5.91	89	152		120	138	168	1.89	293	1	1.25	4.26
^{4}He	^{3}He	^{2}H↓	2	2	1	V[3,4]	5.38	5.01		5.18	5.73	5.59	113	204		162	844	862	7.63	118	1	0.44	12.3
^{4}He	^{3}He	^{3}H↓	2	1	2	V[3,4,9]	5.38		7.28	5.18	6.10	5.91	91		159	125	130	162	1.78	323	1	1.57	3.76
^{4}He	^{3}He	^{3}H↓	1	1	3	V[3,4,9]			7.28	5.18	6.10	5.91			173	168	160	200	1.25	220	0	1.15	4.42
	^{3}He	^{3}H↓		2	3	V[3,4,9]		5.01	7.28			5.91		322	218			282	1.48	89	0	0.44	6.79
	^{3}He	^{3}H↓		1	4	V[4,9]			7.28			5.91			179			239	1.34	217	0	1.23	4.44
^{4}He	^{3}H↓	^{2}H↓	3	1	1	V[3,4]	5.38			6.10	5.73	6.88	85			129	385	409	4.81	323	3	1.49	5.38
^{4}He	^{3}H↓	^{2}H↓	2	2	1	V[3,4,9]	5.38	7.28		6.10	5.73	6.88	115	181		158	401	418	3.63	176	1	0.87	5.89
^{4}He	^{3}H↓	^{2}H↓	2	1	2	V[3,4,9]	5.38		6.58	6.10	5.73	6.88	216		2007	342	1040	1092	9.29	26	1	0.11	21.0
^{4}He	^{3}H↓	^{2}H↓	1	3	1	V[4,9]		7.28		6.10	5.73	6.88		200		187	448	463	2.48	140	0	0.75	6.19
^{3}He	^{3}H↓	^{2}H↓	1	3	1	V[4,9]		7.28		5.91	5.59	6.88		271		396	715	600	2.64	45	0	0.23	9.71
^{4}He	^{3}H↓	^{2}H↓	1	2	2	V[4,9]		7.28	6.58	6.10	5.73	6.88		560	1800	525	1277	1346	3.43	1.5	0	0.0071	24.2
^{4}He	^{3}H↓	^{2}H↓		4	1	V[9]		7.28				6.88		198				398	2.01	179	0	1.06	5.07
^{4}He	^{3}H↓	^{2}H↓		3	2	V[9]		7.28	6.58			6.88		355	1122			758	3.16	28	0	0.15	12.5
^{3}He	^{4}He	^{6}Li	2	2	1	V[3,10]	5.01	5.38		5.18	10.66	11.15	162	88		120	207	172	2.35	338.3[1]	3	4.21	2.02
^{3}He	^{4}He	^{7}Li	2	2	1	V[3,10]	5.01	5.38		5.18	10.79	11.32	154	85		121	190	156	2.24	383.5[1]	3	5.30	1.80
^{3}He	^{4}He	^{23}Na	2	2	1	V[3,10]	5.01	5.38		5.18	11.60	12.34	145	81		113	163	132	2.01	505.1[1]	5	13.7	1.13
^{3}He	^{4}He	^{39}K	2	2	1	V[3,10]	5.01	5.38		5.18	12.85	13.71	150	84		118	203	167	2.42	380.1[1]	3	14.7	1.09
^{3}He	^{4}He	^{40}K	2	2	1	V[3,10]	5.01	5.38		5.18	12.85	13.72	150	84		118	203	166	2.42	381.8[1]	3	14.9	1.08
^{3}He	^{4}He	^{41}K	2	2	1	V[3,10]	5.01	5.38		5.18	12.86	13.72	149	84		118	201	165	2.39	382.9[1]	3	15.0	1.07
^{3}He	^{4}He	^{85}Rb	2	2	1	V[3,10]	5.01	5.38		5.18	13.18	14.11	150	84		118	208	170	2.48	369.3[1]	3	17.4	1.02
^{3}He	^{4}He	^{87}Rb	2	2	1	V[3,10]	5.01	5.38		5.18	13.19	14.12	150	84		118	209	171	2.49	370.1[1]	3	17.5	1.02
^{3}He	^{4}He	^{133}Cs	2	2	1	V[3,10]	5.01	5.38		5.18	13.67	14.65	155	86		121	232	192	2.70	320.6[1]	3	17.1	1.05
^{3}He	^{4}He	^{6}Li	1	3	1	V[3,10]		5.38		5.18	10.66	11.15		71		98	182	153	2.56	605.7[1]	6	7.96	1.59
^{3}He	^{4}He	^{7}Li	1	3	1	V[3,10]		5.38		5.18	10.79	11.32		70		97	172	144	2.46	658.9[1]	6	9.62	1.45
^{3}He	^{4}He	^{23}Na	1	3	1	V[3,10]		5.38		5.18	11.60	12.34		67		95	153	124	2.28	801.9[1]	7	23.4	0.96
^{3}He	^{4}He	^{39}K	1	3	1	V[3,10]		5.38		5.18	12.85	13.71		70		94	185	155	2.64	659.6[1]	6	27.6	0.92
^{3}He	^{4}He	^{40}K	1	3	1	V[3,10]		5.38		5.18	12.85	13.72		69		95	186	154	2.70	661.2[1]	6	27.9	0.92
^{3}He	^{4}He	^{41}K	1	3	1	V[3,10]		5.38		5.18	12.86	13.72		69		95	185	154	2.68	662.8[1]	6	28.2	0.91
^{3}He	^{4}He	^{85}Rb	1	3	1	V[3,10]		5.38		5.18	13.18	14.11		71		95	192	159	2.70	648.2[1]	6	33.4	0.87
^{3}He	^{4}He	^{87}Rb	1	3	1	V[3,10]		5.38		5.18	13.19	14.12		69		96	191	159	2.77	649.2[1]	6	33.6	0.87
^{3}He	^{4}He	^{133}Cs	1	3	1	V[3,10]		5.38		5.18	13.67	14.65		70		96	209	176	2.99	592.3[1]	6	34.6	0.89
	^{4}He	^{6}Li		4	1	V[3,10]		5.38				11.15		63				144	2.29	937.5[1]	10	12.9	1.35
	^{4}He	^{7}Li		4	1	V[3,10]		5.38				11.32		62				136	2.19	997.0[1]	10	15.3	1.24
	^{4}He	^{23}Na		4	1	V[3,10]		5.38				12.34		61				120	1.97	1160.1[1]	10	36.1	0.85
	^{4}He	^{39}K		4	1	V[3,10]		5.38				13.71		62				148	2.39	1001[1]	10	45.1	0.82
	^{4}He	^{40}K		4	1	V[3,10]		5.38				13.72		62				147	2.37	1003.4[1]	10	45.5	0.81
	^{4}He	^{41}K		4	1	V[3,10]		5.38				13.72		62				147	2.37	1004.8[1]	10	45.9	0.81
	^{4}He	^{85}Rb		4	1	V[3,10]		5.38				14.11		63				151	2.40	989.6[1]	10	55.3	0.77
	^{4}He	^{87}Rb		4	1	V[3,10]		5.38				14.12		62				150	2.42	991[1]	10	55.6	0.77
	^{4}He	^{133}Cs		4	1	V[3,10]		5.38				14.65		62				167	2.69	925.5[1]	10	58.8	0.79
		H_2			5	V[11]			7.69						27.8				1.00	44340	10	218	0.470
		^{20}Ne			5	V[12]			11.62						11.1				1.00	224290	10	24970	0.082
		^{40}Ar			5	V[12]			24.62						14.67				1.00	1110100	10	1108500	0.024

References

[1] Stipanović, P. & Vranješ Markić, L. Structure of Helium–Alkali Pentamers. *Few-Body Syst* **59**, 45. https://doi.org/10.1007/s00601-018-1367-x (2018).

[2] Stipanović, P., Vranješ Markić, L., Boronat, J. & Kežić, B. Ground state of small mixed helium and spin-polarized tritium clusters: A quantum Monte Carlo study. *J. Chem. Phys.* **134**, 054509. https://doi.org/10.1063/1.3530837 (2011).

[3] Aziz, R. A., McCourt, F. R. W. & Wong, C. C. K. A new determination of the ground state interatomic potential for He2. *Molecular Physics* **61**, 1487–1511. https://doi.org/10.1080/00268978700101941 (1987).

[4] Chung, H.-K. & Dalgarno, A. Diffusion of hydrogen atoms in helium gas and helium atoms in hydrogen gas. *Phys. Rev. A* **66**, 012712. doi.org/10.1103/PhysRevA.66.012712 (2002).

[5] Meyer, W. & Frommhold, L. Long-range interactions in H-He:ab initio potential, hyperfine pressure shift and collision-induced absorption in the infrared. *Theoret. Chim. Acta* **88**, 201–216. https://doi.org/10.1007/BF01113614 (1994).

[6] Das, G., Wagner, A. F. & Wahl, A. C. Calculated long-range interactions and low energy scattering in He+H, Ne+H, Ar+H, Kr+H, and Xe+H. *J. Chem. Phys.* **68**, 4917–4929. https://doi.org/10.1063/1.435648 (1978).

[7] Tang, K. T. & Yang, X. D. Interaction potential of the H-He system and the hyperfine frequency shift of H in He buffer gas. *Phys. Rev. A* **42**, 311–319. https://doi.org/10.1103/PhysRevA.42.311 (1990).

[8] Toennies, J. P., Welz, W. & Wolf, G. The determination of the H-He potential well depth from low energy elastic scattering. *Chemical Physics Letters* **44**, 5–7. https://doi.org/10.1016/0009-2614(76)80396-X (1976).

[9] Jamieson, M. J., Dalgarno, A. & Wolniewicz, L. Calculation of properties of two-center systems. *Phys. Rev. A* **61**, 042705. https://doi.org/10.1103/PhysRevA.61.042705 (2000).

[10] Kleinekathöfer, U., Lewerenz, M. & Mladenović, M. Long Range Binding in Alkali-Helium Pairs. *Phys. Rev. Lett.* **83**, 4717–4720. https://doi.org/10.1103/PhysRevLett.83.4717 (1999).

[11] Silvera, I. F. & Goldman, V. V. The isotropic intermolecular potential for H2 and D2 in the solid and gas phases. *J. Chem. Phys.* **69**, 4209–4213. doi.org/10.1063/1.437103 (1978).

[12] Tang, K. T. & Toennies, J. P. The van der Waals potentials between all the rare gas atoms from He to Rn. *J. Chem. Phys.* **118**, 4976–4983. https://doi.org/10.1063/1.1543944 (2003).
